# T-Cell Subsets in Rheumatoid Arthritis Patients on Long-Term Anti-TNF or IL-6 Receptor Blocker Therapy

**DOI:** 10.1155/2017/6894374

**Published:** 2017-10-25

**Authors:** Sonja Dulic, Zsófia Vásárhelyi, Florentina Sava, László Berta, Balázs Szalay, Gergely Toldi, László Kovács, Attila Balog

**Affiliations:** ^1^Department of Rheumatology and Immunology, Faculty of Medicine, Albert Szent-Györgyi Health Center, University of Szeged, Szeged, Hungary; ^2^First Department of Obstetrics and Gynecology, Semmelweis University, Budapest, Hungary; ^3^First Department of Pediatrics, Semmelweis University, Budapest, Hungary; ^4^Department of Laboratory Medicine, Semmelweis University, Budapest, Hungary

## Abstract

Data on the impact of biological therapies on the T-cell phenotype in rheumatoid arthritis are limited. Here, we prospectively measured the percentages of 15 circulating T-cell subtypes using flow cytometry. We obtained transversal and longitudinal data in 30 anti-TNF responders, 19 secondary anti-TNF nonresponders, and 43 IL-6R antagonist responders, before, 8 weeks and at least 6 months after biological therapy. Untreated RA patients and healthy controls were also included. The important findings are the following: (1) the proportion of regulatory T-cells (Tregs) which are decreased in untreated RA patients becomes normal in all long-term-treated groups; (2) in anti-TNF responders as well as in nonresponders, the frequencies of naïve CD4+ and CD8+ cells are lower, whereas those of proinflammatory Th1, Th2, and Th17 cells and HLA-DR+-activated cells are higher than those in untreated RA or healthy controls; (3) in IL-6R responders, Th1 proportion is decreased, while that of Th2 and Th17 is increased as compared to that in anti-TNF-treated patients and controls; (4) pending confirmation, a CD4CD69 ratio < 2.43 at baseline, could be useful to predict a good therapeutic response to anti-TNF therapy. This study provides comprehensive information regarding the long-term impacts of those biological therapies on the ecotaxis of T-cells in RA. The ClinicalTrials.gov registration number of our study is NCT03266822.

## 1. Introduction

Rheumatoid arthritis (RA) is the most common chronic autoimmune joint disease, which leads to progressive articular destruction without treatment [1]. The abnormal function of CD4+ and CD8+ cells plays a key role in the autoimmune process leading to the development of RA. This is reflected by a number of observations indicating that the proportion of different CD4+ subsets responsible for the harmonized immune response is skewed to a proinflammatory direction. The frequency of Th1, Th2 helper, and proinflammatory Th17 cells is increased [2, 3], while that of regulatory T-cells (Treg) is decreased in the peripheral blood of RA patients [4–7]. Biological therapies, including monoclonal antibodies targeting tumor necrosis factor-*α* (TNF) and interleukin-6 receptor (IL-6R), have emerged as disease-modifying agents with much higher therapeutic potential than conventional immunosuppressive therapies. Little is known about how the alterations in the T-cell subset composition are affected by anti-TNF or anti-IL-6R drugs. Few studies, including our previous examinations [7], followed T-cell subset prevalence changes, but in most of them, only short-term follow-up was evaluated [8–15]. As changes in cell numbers are supposed to require longer time, we presume that short-term follow-up may not be sufficient. Furthermore, the number of patients was not high enough to capture subtle changes in cell proportions; moreover, some studies were not homogenized for disease activity or response to therapy, or only few types of cells were monitored. Data on the effects of IL-6R blocker therapy are especially limited [16–18].

Our knowledge about the long-term consequences of biological therapies is still insufficient. Data on the risk of the susceptibility to infections, efficacy of vaccination, or tumor development after several years of anti-TNF therapy are not yet conclusive [19, 20]. A detailed insight into how a sustained interference to the adaptive immune system with biological therapies skews the status of the adaptive immune system would provide useful information in this regard. Furthermore, as only about 40% of patients respond with complete remission to anti-TNF or anti-IL-6R treatment, and the number of available therapies with different target specificities is increasing, there is a highly recognized need for predictors of a good response for every therapeutic agent to establish the choice of therapy in a personalized manner. Although some soluble predictive biomarkers have been proposed [21, 22], predictors relating to the cellular component of the immune system, as identified through a long-term follow-up assessment, are lacking.

We aimed to answer the following questions: (1) Is the T-cell subset distribution different in RA patients on long-term (more than six-month duration) biological therapy as compared to the short-term data (baseline, i.e., biological therapy naive patients and short term: eight-week anti-TNF therapy)? (2) Is the immune phenotype different between anti-TNF responder and nonresponder patients? and (3) Are there any T-cell subtypes that can be used as predictors of the response to anti-TNF therapy? Finally, we wished to analyze the T-cell phenotype in patients on IL-6R blocker therapy.

Herein, we present a detailed description of the T-cell phenotype of RA patients on established biological therapies, obtained with two approaches: (1) a cross-sectional analysis of a high number of RA patients on a long-term treatment with anti-TNF or anti-IL-6R therapies; (2) we present the long-term follow-up results of our prospective study of anti-TNF-treated RA patients, in whom these parameters have serially been measured from the start of the anti-TNF treatment (short-term follow-up data have been published in [7]). The evaluation of the long-term outcome of anti-TNF therapy enabled us to evaluate which T-cell subset changes may be predictive of a long-standing therapeutic response to these treatment agents.

## 2. Patients and Methods

### 2.1. Patients

In the cross-sectional analysis, 92 RA patients (who had been treated with biological therapy for more than six months) were evaluated. All of them are treated at the Department of Rheumatology and Immunology, University of Szeged. Rheumatoid arthritis was classified according to the 2010 ACR/EULAR classification criteria for RA [23]. 49 patients were treated with anti-TNF therapy (adalimumab *n* = 14, certolizumab pegol *n* = 14, etanercept *n* = 11, infliximab *n* = 6, and golimumab *n* = 4 patients) and 43 patients with the anti-IL-6R agent tocilizumab. Within the anti-TNF-treated RA patients, we distinguished anti-TNF responder or anti-TNF nonresponder patients. For the definition of the therapeutic response, we used the EULAR good response criteria [24]; therefore, in the responder group, patients had a DAS28 score of ≤3.2, and its improvement since the initiation of the biological therapy was >1.2. Since there were insufficiently low numbers of IL-6R antagonist nonresponder patients within our patient population, we included only IL-6R blocker responders in this study.

We compared their results with newly diagnosed, untreated RA patients (*n* = 19). Treatment-naïve, early RA patients had not received any anti-RA treatment prior to our study. The measurements on this cohort of patient have been published earlier [7]. The detailed clinical data and patient characteristics are presented in Table 1. ACPA was measured using ELISA-based routine laboratory methods with specificity to mutated citrullinated vimentin.

As a further control group (healthy controls), we enrolled 30 age- and gender-matched healthy volunteers (18 men, 12 women; mean age 52.4 years range (24–63.5). All of them had a negative history of RA symptoms and a negative status upon detailed physical and laboratory examination including normal CRP and ESR values.

The prospective follow-up cohort included 13 of the anti-TNF-treated patients, in whom T-cell frequency values at the initiation of anti-TNF therapy and 8 weeks thereafter were measured earlier [7]. Six of them have become (secondary) nonresponders in the long-run (i.e., since the completion of the short-term follow-up), whereas 7 of them remained to be long-term responders to anti-TNF therapy. Average age of these 13 patients was 59 (39–65) years, and the mean duration of anti-TNF treatment was 27 (11–52) months; there was no statistically significant difference between the two groups in either of these parameters. The conditions of the present laboratory measurements and all the clinical assessments were fully identical to those described for the study on therapy-naïve patients and those on short-term anti-TNF therapy [7].

Informed consent was signed by all participants, and the protocol had been approved by the Ethics Committee of the Ministry of Health of Hungary and Ethics Committee of the University of Szeged (ETT-TUKEB905/PI/09).

### 2.2. Flow Cytometry Measurements

Blood samples were taken in the laboratory unit of the Department of Rheumatology and Immunology. We used 15 ml of anticoagulated blood and separated the peripheral blood mononuclear cells (PBMCs) by centrifugation with Ficoll-Paque (GE Healthcare Life Sciences, Pittsburgh, PA, USA). PBMCs were frozen and kept at −80°C until analysis. After thawing, samples were washed twice with phosphate-buffered saline solution (pH 7.4). We used fluorescent antibodies (Becton Dickinson, San Diego, CA, USA) for cell surface staining according to the manufacturer's manual.

**Figure 1:**
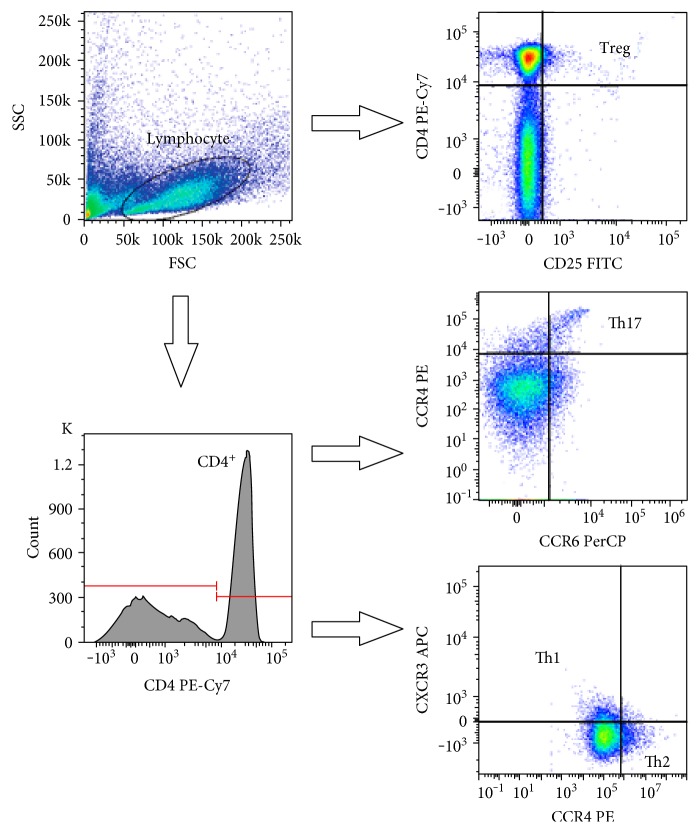
Gating strategy for the identification of T-helper cell subsets with flow cytometry. FSC: forward scatter characteristics; SSC: side scatter characteristics.

Cell subtypes were defined as helper T-cells (CD4+), Th1 cells (CD4+CXCR3+), Th2 cells (CD4+CCR4+CCR6−), Th17 cells (CD4+CCR4+CCR6+), Tregs (CD4+CD25 high), naive T-cells (CD4+CD45RA+), or memory T-cells (CD4+CD45RO+). The proportion of cells expressing early (CD69), intermediate (CD25), or late (HLA-DR) activation markers was also determined within both the CD4+ and CD8+ subsets. An average of 200,000 cells were registered for each acquisition. All measurements were performed on a BD FACSAria flow cytometer (Becton Dickinson, San Jose, CA, USA). Cell proportion values were determined with conventional gating, through the use of FACSDiva software (Becton Dickinson, San Jose, CA, USA).  Figure 1 represents the gating strategy of T-helper subsets.

### 2.3. Statistical Methods

Data are presented as mean ± SD or median (25–75 percentile) depending on the distribution of the values. Cell subset percentage values were compared among groups with analysis of variance or with Kruskal-Wallis test, with Bonferroni's or Dunn's tests for multiple comparisons, respectively. Predictive value of baseline percentage values to subsequent response to anti-TNF therapy was assessed with ROC analysis. A *p* value < 0.05 was taken as statistically significant.

## 3. Results

### 3.1. Patient Characteristics

Thirty of the 49 anti-TNF RA patients were responders and 19 were nonresponders, while all of the 43 IL-6R-treated RA patients were responders. As it can be seen in Table 1, mean ages, disease duration, and the proportion of patients who were on low-dose corticosteroid therapy in these three groups were not different. Fewer IL-6R responders took traditional DMARDs than the anti-TNF-treated patients, and the proportion of anti-citrullinated peptide antibody- (ACPA-) positive patients was also lower in the IL-6R blocker-treated group. The newly diagnosed untreated RA patients were slightly younger than the long-term-treated patients; all of them were ACPA-positive, and they had the highest mean DAS28 score.

### 3.2. Immunophenotype of Patients with RA on Long-Standing Anti-TNF Therapy

**Figure 2:**
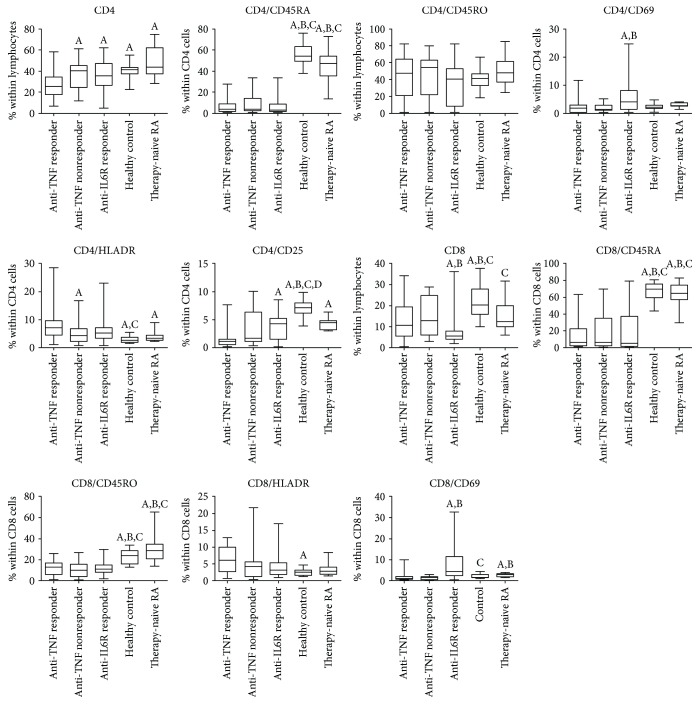
Proportions of various T-cell subsets in rheumatoid arthritis patient groups of anti-TNF responders (*n* = 30), anti-TNF nonresponders (*n* = 19), and IL-6R blocker responders (*n* = 43), early, active, therapy-naïve RA patients (*n* = 19), and healthy controls (*n* = 30). Data are presented as median (horizontal line within boxes), 25 and 75 percentile (horizontal borders of the boxes), and minimum and maximum (whiskers). ^A^*p* < 0.05 versus anti-TNF responder, ^B^*p* < 0.05 versus anti-TNF nonresponder, ^C^*p* < 0.05 versus IL-6R blocker responder, ^D^*p* < 0.05 versus early, untreated RA patients.

**Figure 3:**
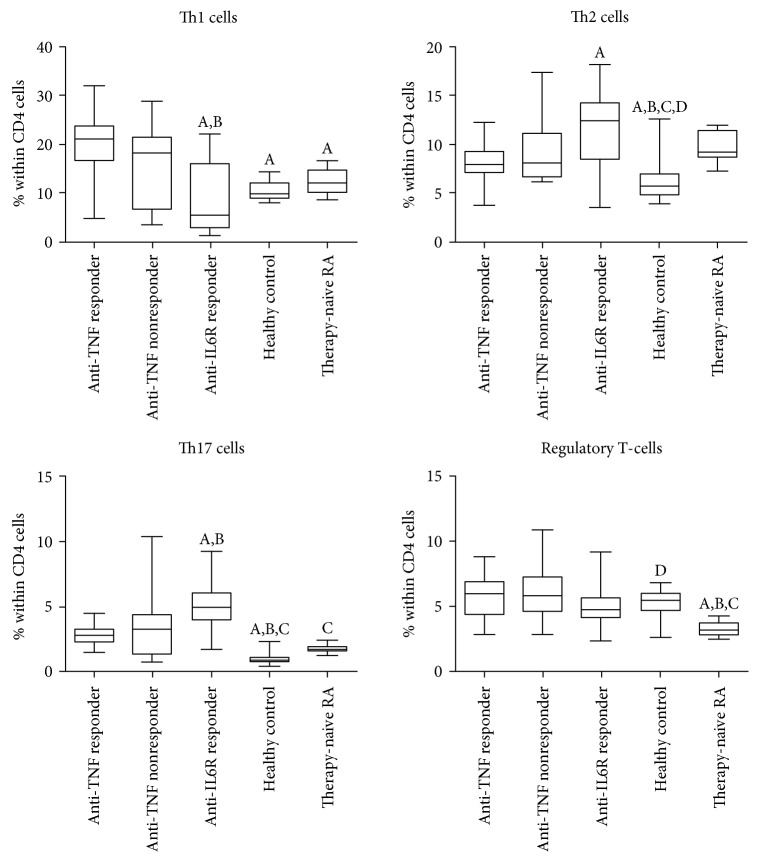
Proportions of the major effector T-helper cell subsets in rheumatoid arthritis patient groups of anti-TNF responders (*n* = 30), anti-TNF nonresponders (*n* = 19), and IL-6R blocker responders (*n* = 43), early, active, therapy-naïve RA patients (*n* = 19), and healthy controls (*n* = 30). Data are presented as median (horizontal line within boxes), 25 and 75 percentile (horizontal borders of the boxes), and minimum and maximum (whiskers). ^A^*p* < 0.05 versus anti-TNF responder, ^B^*p* < 0.05 versus anti-TNF nonresponder, ^C^*p* < 0.05 versus IL-6R blocker responder, ^D^*p* < 0.05 versus early, untreated RA patients.

The proportions and ratios of various T-cell subsets are demonstrated in Table 2 and Figures 2 and 3.

### 3.3. Anti-TNF Responders

As compared with early, active, untreated RA, anti-TNF responders had lower proportions of CD4+ cells, naïve CD4+ and CD8+ cells and memory CD8+ cells, and higher percentages of activated CD4+ T-cells with HLADR marker positivity, but lower prevalences of CD4+ cells with CD25 and of CD8+ T-cells with CD69 marker positivity. Anti-TNF responders were characterized by higher Th1 and Treg frequencies than early active, untreated RA patients.

When compared with healthy controls, anti-TNF responders had lower proportions of CD4+ and CD8+ T-cells. The frequencies of naive T-cells (both CD4+ and CD8+CD45RA+ cells) were lower compared with controls, whereas those of the memory subtype (CD45RO+) were similar among CD4+ cells and were also lower among CD8+ cells in the anti-TNF responders than in healthy volunteers. The proportion of activated T-cells bearing the CD25 marker was lower and that of the HLA-DR+ cells (both CD4+ and CD8+) was higher in anti-TNF responders than in controls (Figure 2). In anti-TNF responders, Th1, Th2, and Th17 proportion values were all higher than those in healthy controls, but, importantly, Treg frequencies were not different (Figure 3).

### 3.4. Anti-TNF Nonresponders

There are somewhat less differences between the T-cell composition of anti-TNF nonresponders and early untreated RA patients, but naïve T-cells (both CD4+ and CD8+) and also CD8+ memory cells are less prevalent in anti-TNF nonresponders, similarly to CD8CD69+-activated cells. A comparison with healthy controls also revealed that anti-TNF nonresponders had lower proportions of CD4+ and CD8+ naïve and CD8+ memory T-cells; furthermore, CD4+CD25+-activated T-cells also occurred less frequently in the anti-TNF nonresponders than in the healthy subjects. The percentage of CD8+ cells was lower in anti-TNF nonresponders than in controls, but, in contrast with anti-TNF responders, CD4+ cell prevalence was not different from controls (Figure 2). Similarly to the anti-TNF responders, Th17 and Th2 percentages were also higher in nonresponders than in the controls, and, again, Treg frequencies were equal to the healthy controls (Figure 3).

The differences between anti-TNF responders and nonresponders, as revealed in this analysis, were significantly lower percentages of total CD4+ and higher proportion of CD4+HLA-DR+ T-cells in anti-TNF responders as compared with anti-TNF nonresponders.

### 3.5. Immunophenotype of Patients with RA on Long-Standing IL-6 Receptor Blocker Therapy

Most important differences between the T-cell composition of RA patients on effective IL-6R blocker therapy and early, active, untreated RA patients are the strikingly low number of CD8+ cells and the higher prevalence of Th17 and Treg cells in the IL-6R blocker-treated patients. Naive T-cell (both CD4+ and CD8+) and CD8+ memory cell proportions were lower in anti-IL-6R-treated RA patients (Figures 2 and 3). As compared with healthy subjects, some further differences can also be observed: higher prevalence of Th2 cells and of CD4+HLA-DR+- and CD8+CD69+-activated T-cells than in controls.

Comparisons between anti-IL-6R responders and anti-TNF responders reveal significantly higher CD4+ and lower CD8+ T-cell frequencies with anti-IL-6R therapy (Figure 2). Anti-IL-6R responders had the lowest proportion of Th1 cells in all the examined groups, and this difference was significant from both anti-TNF responders and anti-TNF nonresponders (Figure 3). On the contrary, the proportions of Th2 and Th17 cells were higher among anti-IL-6R responders than in anti-TNF-treated RA patients including anti-TNF responders and anti-TNF nonresponders. Nevertheless, similarly to anti-TNF-treated groups, Treg frequencies were normal (Figure 3). With regards to the activated T-cell subsets, anti-IL-6R therapy was associated with higher percentages of CD69+ T-cells, within both the CD4+ and the CD8+ subsets, than anti-TNF therapy, and CD4+CD25+ cells were also more prevalent in anti-TNF responders (Figure 2).

### 3.6. Time-Course of the Changes in the T-Cell Subset Distribution in RA Patients on Long-Standing Anti-TNF Therapy

**Figure 4:**
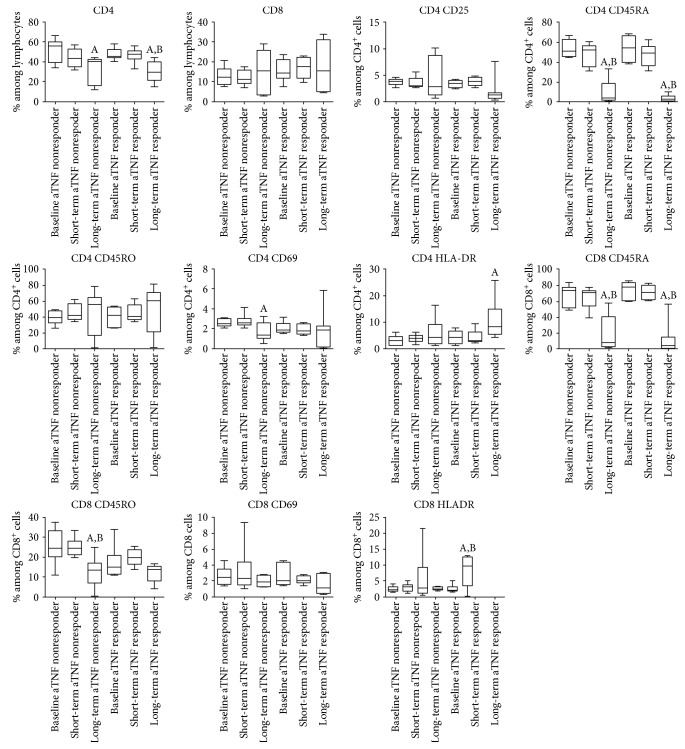
Changes in the proportions of T-cell subsets in RA patients in whom long-term follow-up data from the initiation of anti-TNF therapy were available (*n* = 13). 7 patients from the initial short-term cohort (7) proved to be long-term responders, whereas the other 6 patients lost the initial response and therefore belonged to the nonresponder group. Measurement time points: baseline: at anti-TNF initiation, short term: 8 weeks of anti-TNF treatment (previously published data (7)), long-term: current measurement results after long-standing anti-TNF treatment. Data are presented as median (horizontal line within boxes), 25 and 75 percentile (horizontal borders of the boxes), and minimum and maximum (whiskers). ^A^*p* < 0.05 versus baseline, ^B^*p* < 0.05 versus short term.

**Figure 5:**
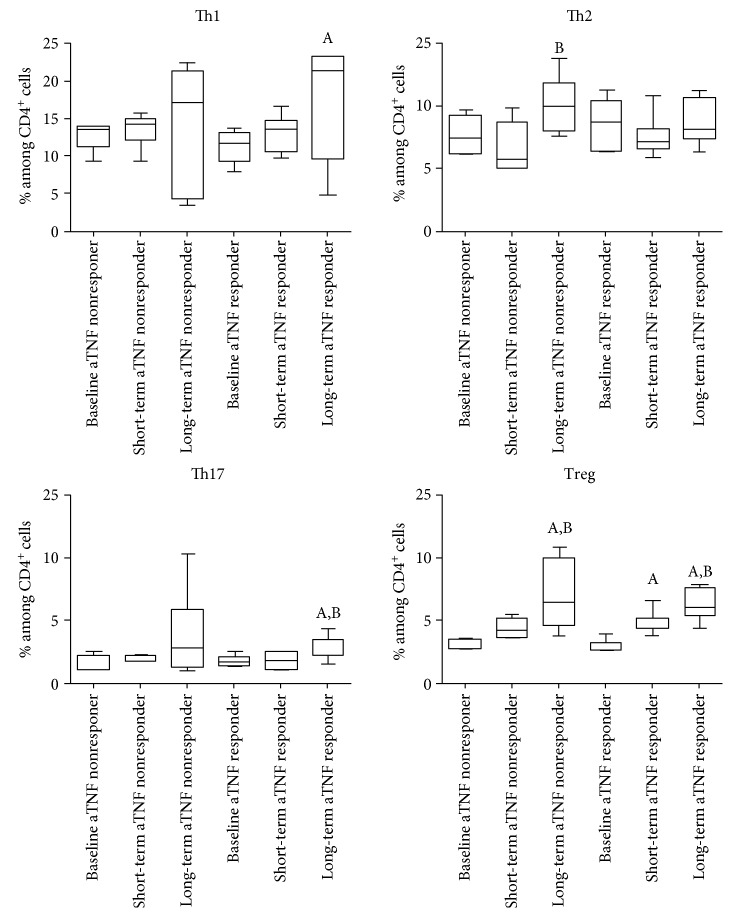
Changes in the proportions of the major effector T-helper cell subsets in RA patients in whom long-term follow-up data from the initiation of anti-TNF therapy were available (*n* = 13). 7 patients from the initial short-term cohort (7) proved to be long-term responders, whereas the other 6 patients lost the initial response and therefore belonged to the nonresponder group. Measurement time points: baseline: at anti-TNF initiation, short term: 8 weeks of anti-TNF treatment (previously published data (7), long-term: current measurement results after long-standing anti-TNF treatment. Data are presented as median (horizontal line within boxes), 25 and 75 percentile (horizontal borders of the boxes), and minimum and maximum (whiskers). ^A^*p* < 0.05 versus baseline, ^B^*p* < 0.05 versus short term.

We compared the T-cell subset proportion values from the beginning of the disease in 13 patients (7 anti-TNF responders and 6 anti-TNF nonresponders). As compared with the baseline values (at disease onset, before anti-TNF therapy initiation), percentages of total CD4+ T-cells, CD4+ and CD8+ naive T-cells decreased (Figure 4), but those of Tregs increased over time in both anti-TNF responders and anti-TNF nonresponders (Figure 5). Th1 and Th17 proportions increased only in the anti-TNF responder group, and Th2 cell frequencies increased only within the anti-TNF nonresponders (Figure 5). CD4+CD69+ cell proportion decreased in the anti-TNF nonresponders (*p* < 0.05 with ANOVA, but no significant differences with Bonferroni's correction), and CD4+HLA-DR+ cell percentages increased only in the anti-TNF responders (Figure 5). Among CD8+ cells, memory T-cells became less prevalent during the course of the disease only in the anti-TNF nonresponders, while HLA-DR+-activated cell frequency was gradually rising in the anti-TNF responder group only. As it can be seen in Figures 4 and 5, most of these changes have become evident only after long-term follow-up.

### 3.7. The Impact of the Length of the Biological Therapy on T-Cell Subsets in Long-Term-Treated RA Patients

Since the duration of biological therapies was highly variable among long-term-treated RA patients (ranging from 6 to 52 months), the question may arise whether this wide time span could have an impact on the long-term effects of biologicals on the T-cell composition. We therefore further stratified the patients according to the duration of long-term biological therapy to “short” (6–12 months), “medium” (12–18 months), and “long” (>18 months) duration of treatment. Comparison among these subgroups has revealed that only two of the examined 15 T-cell subtypes displayed a significant variability across these three subgroups: the prevalences of CD4+CD45RA+ and CD8+CD45RA+ naive cells decrease gradually among the three subgroups with longer treatment duration and reach a significance of *p* < 0.05 in the comparison between long-term duration versus the other two subgroups. All the other parameters have remained stable irrespective of the length of biological therapy (Table 3).

### 3.8. The Impact of Previous Switching of Anti-TNF Agents on T-Cell Subsets

**Figure 6:**
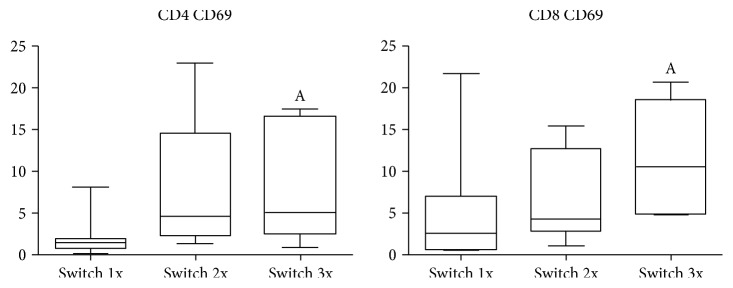
The frequencies of CD4+CD69+ and of CD8+CD69+ cells in anti-IL-6R responder RA patients grouped according to the number of previous switches in anti-TNF therapy before the initiation of the present tocilizumab treatment. Numbers indicate percentages within the CD4+ or CD8+ T-cells, as applicable. ^A^*p* < 0.05 versus 1x switch.

As it can be seen in Table 1, in some patients (especially in the IL-6R blocker-treated group), the biological agent applied at the time of sampling was not the first one, but there were previous switches from other anti-TNF drugs. Although these switches occurred more than 6 months before the blood sampling, we wanted to know whether the previous changes in therapy may have influenced the T-cell phenotype. We therefore compared the patient subgroups as defined by the number of previous switches in all therapeutic groups (Table 4). This analysis revealed that the proportions of CD4+CD69+ and of CD8+CD69+ cells were higher in IL-6R responder patients who had experienced three switches before the current therapy than in those who had only one switch before (Figure 6). Opposite difference was observed with regard to Th1 percentage.

### 3.9. Differences among the Various Anti-TNF Agents

When we compared the T-cell subset proportions among the 5 anti-TNF agents individually (including adalimumab, etanercept, certolizumab, golimumab, and infliximab) and between the particular anti-TNF drugs and anti-Il-6R responders or healthy controls, only one significant difference was revealed in addition to the comparisons when the anti-TNF drugs were considered as one single group: the frequencies of CD4+CD45RO+ cells were higher among etanercept-treated patients (responders and nonresponders taken together) than among IL-6R blocker responders. The effect of the various anti-TNF agents on the immunophenotype of the RA patients was not different (Table 5).

### 3.10. Relationship between Baseline T-Cell Subset Prevalences and Response to Therapy

**Figure 7:**
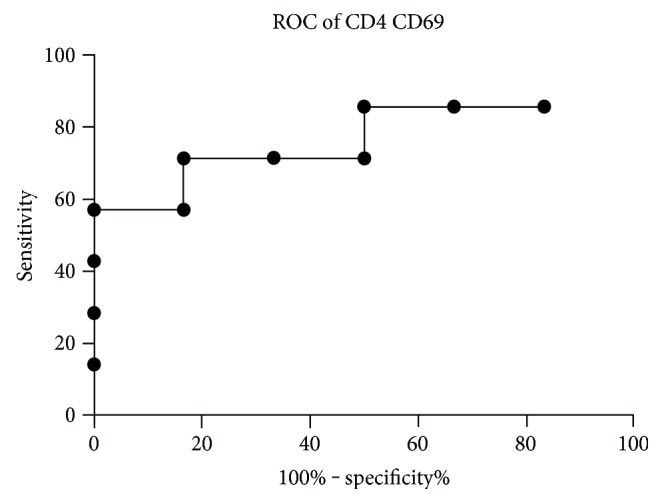
Receiver-operator characteristic (ROC) curve of CD4CD69 positivity. Predictive potential of the percentage of CD4CD69-positive cells at baseline (start of anti-TNF) to long-term response to therapy. CD4+CD69+ T-cell percentage < 2.43 has the highest likelihood ratio (4.29 (CI: 0.58–1.06) to discriminate between future anti-TNF responders and nonresponders (sensitivity: 71.4%, specificity: 83.3%, *p* = 0.054).

Through the analysis of the prospective follow-up cohort, in which we compared the T-cell subset frequencies at baseline (i.e., at the start of the anti-TNF therapy), short-term (8 weeks), and long-term therapies, we examined whether any baseline parameters are predictive of the long-term response to anti-TNF therapy. The proportion of CD4+CD69+ T-cells at baseline (2.16 ± 0.12 versus 2.69 ± 0.16, *p* = 0.08) and at 8 weeks (2.01 ± 0.20 versus 2.81 ± 0.28, *p* = 0.03) was lower in those who later belonged to anti-TNF responders than in those who became anti-TNF nonresponders. ROC analysis revealed that a CD4+CD69+ T-cell percentage < 2.43 at baseline predicts a future response to anti-TNF therapy with a likelihood ratio of 4.29 (CI: 0.58–1.06) and discriminates between future anti-TNF responders and nonresponders with a sensitivity of 71.4% and a specificity of 83.3% (*p* = 0.054) (Figure 7).

## 4. Discussion

Our results present a comprehensive overview of the alterations in the composition of the T-cell subset in RA patients on long-term anti-TNF or IL-6R blocker therapy with a focus on changes in the naive/memory subtypes, the most important effector pathways (Th1, Th2, Th17, and Treg), as well as various activation markers (CD25, CD69, and HLA-DR). Key findings are that, during anti-TNF therapy, the reduced percentages of Tregs found in active, untreated disease gradually normalize, while the proportion of naive T-cells decreases, and, surprisingly, the proportions of Th1 and Th17 cells, which are important drivers of RA activity, also remain increased and even further rise with follow-up. Some of these alterations were dependent on the therapeutic response, whereas many of them seemed to be a characteristic effect of anti-TNF therapy independent of its disease-controlling effect.

There are a few reports about T-cell subset changes during anti-TNF therapy; most of them involve relatively low numbers of patients [8, 9, 12, 13], use only short-term follow-up [7, 8, 11, 12], and the majority are restricted to the determination of Treg and Th17 proportions. Although most of the studies describe an increase in Treg and a decrease in Th17 frequencies [8, 11, 12, 14], opposing results have also been published [7, 10, 15]. Chen et al. found that Th17 cell counts decrease in anti-TNF-treated patients who had shown a good response to therapy, whereas in nonresponders, Th17 cell percentages increased [14]. In our previous study involving only 8 weeks of short-term follow-up, we have demonstrated that Th1 and Treg frequencies increased compared with the values measured at the time of the initiation of anti-TNF therapy, but the values remained significantly different from those of healthy controls [7]. With extended follow-up, the difference has disappeared relating to Tregs, whereas other alterations have remained or new ones have developed. The ratio of naïve T-cells decreased, that of Th1 or Th17 cells increased, CD4+CD69 cell counts decreased, and CD4+HLA-DR+ cells increased, and some of these changes were different between responders and nonresponders. The discrepancies among the published results may be explained by differences in the follow-up time, the surface markers used, or the patient populations. Based on our previous and present data, we conclude that short-term follow-up is not suitable to capture the T-cell subset alterations occurring during anti-TNF or IL-6R therapy and that changes within the T-cell composition probably progress continuously despite the decrease in the inflammatory activity during treatment.

The increase of Th17 on both types of therapies and of Th1 on anti-TNF therapy, as well as of various activated T-cell subtypes, may be explained by the fact that both anti-TNF and anti-IL-6R therapies exert their action by the inhibition of the terminal phase of the effector arm of the autoimmune process (i.e., cytokine action), and the differentiation and the activation of naive T-cells by the permanent antigenic stimuli may be left unchanged. The elevation of the proportion of T-cells bearing the late activation marker HLA-DR, the decrease of CD4+CD25+ intermediate activation marker-positive cells, and the fact that these changes were more evident among anti-TNF responders than in nonresponders also support this hypothesis. In this regard, it would be interesting to examine the T-cell proportion changes during B-cell depleting or costimulation inhibiting therapies, which influence the afferent phase of the immune response. On the other hand, we have found that Treg frequency gradually normalizes during both TNF- and IL-6R-blocking therapies. While patients with early, untreated active RA display markedly low Treg proportions, both classes of biological therapies seem to restore this abnormality. The increase in Treg proportion starts soon after the initiation of anti-TNF-therapy, but the changes become statistically significant only after long-term treatment. Restoration of tolerance, as shown by the normalization of Treg numbers, seems to be a consistent phenomenon during the prolonged therapy of RA. As the frequencies of many other T-cell subtypes remained different from healthy controls, irrespective of therapy-response, we presume that anti-TNF or IL-6R blocker therapies do not exert their pharmacological actions through the influence on the T-cell subsets other than Tregs, but rather by reducing the levels of acute phase reactants, or synovial cytokine or chemokine production. A further factor that should be considered is that despite Th17 proportion remained increased, the ratio of Th17/Treg tended to approach the ratio observed in healthy controls. Analogously to our findings, Teniente-Serra et al. have found that multiple sclerosis patients treated with natalizumab (monoclonal antibody to the integrin CD49d) had an increased percentage of early effector and central memory T-cells, as well as of early thymic emigrant T-cells, as compared with untreated patients, indicating that T-cell activation may proceed despite the inflammatory process is blocked by the inhibition of the effector steps [25].

The dynamics of the transformation of the T-cell pool can be estimated from our studies: there are relatively great differences between the T-cell phenotype at 8 weeks and 6 months of biological therapy, whereas only minor changes (i.e., further increase in naïve T-cells) occur after 6 months. It indicates that a few months of follow-up are insufficient to address this question, but 6 months is suggested to be a relevant measurement point for T-cell subtype analysis, validating our definition of long-term treatment of at least 6 months.

RA patients on effective long-term tocilizumab therapy in our study are characterized by normal Treg numbers, increased Th2 and Th17 cell proportion, and by decreased frequencies of CD8 and naive (both CD4+ and CD8+) cells. Th1 cell percentages were lower but those of CD69+-activated CD4+ and CD8+ cells were higher than anti-TNF-treated patients. This finding confirms our hypothesis that different classes of targeted therapies have different impacts on the T-cell homeostasis although their efficacy and tolerability as assessed by clinical and routine laboratory examinations are identical. The proportions of various lymphocyte subsets during IL-6R blocker therapy were followed by Kikuchi et al. [16]. Our results add further knowledge to their results by including several T-cell subtypes not analyzed before and the inclusion of a healthy control group. Common findings in their examinations and ours are the importance of the normalization of Treg counts, the decrease in the frequencies of CD8+ cells and naive T-cells, and an increase in those of CD69+-activated T-cells. Although they did not find a significant change in Th1, Th2, or Th17 prevalences, their results also underline that a decrease in the frequencies of these activated T-cell subsets is not a finding that can be expected during tocilizumab treatment.

High body of data confirm that biological therapies can, in general, be regarded as safe also when administered for several years, but the label of each agent warns that their administration in patients with previous malignancy needs individual risk assessment and caution. It was also suggested that long-term rituximab therapy, another type of biological with specificity to B-cells targeting CD20, produces T-cell alterations similar to immunosenescence characterized by an increased susceptibility to infections [26] and that JC virus reactivation causing progressive multifocal leukoencephalopathy may be preceded by specific T-cell subset alterations in natalizumab-treated multiple sclerosis patients [27]. Long-term cohort studies, and especially registry data, should be paralleled with T-cell phenotype analyses to discern whether a sustained decrease of CD8-positive (cytotoxic) T-cells and Th1 cells may impair the protection against carcinogenesis or the reactivation or acquisition of some types of infection.

The most important activation molecules expressed on T lymphocytes can be classified as early activation markers, such as CD69 and CD25, and late activation markers, such as HLA-DR. CD69 is generally regarded as the earliest activation cell surface marker induced by a mitogenic stimulus. The expression of CD69 molecule is not restricted to activated lymphocytes, as activated neutrophils and eosinophils can also express CD69. Moreover, platelets, epidermal Langerhans cells, and bone marrow myeloid precursors express CD69 constitutively. The engagement of CD69 can activate NK and T-cells, resulting in increased cytotoxic activity and proinflammatory cytokine production [28]. CD25, or the alpha subunit of the IL-2 receptor, is involved in the early stage of lymphocyte activation, but it also seems to be critical in maintaining self-tolerance and immune homeostasis. Early work on CD4+CD25 high+ cells later termed as regulatory T-cells showed that their activation via their T-cell receptor (TCR) generates suppressor cells that are capable of nonspecifically suppressing the activation of CD4+ or CD8+ T-cells [29]. HLA-DR molecules are involved in antigen processing and presentation, mediating antigen-specific T-cell activation. They are not expressed by naïve T-cells, but their expression is induced during T-cell activation, driven primarily by class-II transactivator, through the activation of its promoter by CREB/ATF or AML/Runx transcription factors [30]. During the follow-up analysis of anti-TNF responder and nonresponder patients, the early activation marker CD69 emerged as the most useful predictive marker of medication response. If the proportion of CD4+CD69+ cells is lower than 2.43 in an RA patient at the start of anti-TNF therapy, the patient has a high likelihood to show a good response to this biological treatment. This correlation is further supported by our analysis of IL-6R responder patients with previous anti-TNF switch, because in those who had three times produced an incomplete response to anti-TNF therapy previously. The prevalences of CD4CD69+ and CD8CD69+ markers are significantly higher as compared to those who failed only one anti-TNF inhibitor. These results confirm not only the potential predictive role of the low expression of CD4CD69 marker for a good therapeutic response to anti-TNF therapy, but also aid in the drug choice after an incomplete response to a TNF-inhibitor. Since all anti-TNF nonresponders who had previously switched three times and were characterized by higher CD4CD69 and CD8CD69 expressions became long-term anti-IL-6R responders, we propose that for patients with high CD4CD69 expression, after an incomplete TNF-inhibitor treatment, a switch to anti-IL-6R therapy is the preferred choice, although it has to be noted that we do not have information on the percentage of CD4CD69+ cells at the initiation of IL-6R blocker in our patients, and consequently its predictive power needs further confirmation.

Whereas several novel biochemical or clinical biomarkers that aid in the differential diagnoses, disease subset definition or the prediction of the progression of irreversible organ damage has recently been identified [31–33]. There is substantial deficiency in the availability of predictors of the response to immunosuppressive treatment in general, or to particular therapeutic agents. Since almost all chronic inflammatory rheumatic diseases are regarded as heterogeneous syndromes with substantial genetic variability among patients with the same diagnoses, and this genetic heterogeneity renders most of the therapeutic agents effective only in a subset of patients, analysis of susceptibility factors to drug response and adverse effects is crucial. As the paradigm of early, effective, and targeted interventions has become a general requirement in order to prevent early tissue damage and to modify the course of the disease in the long run [34–36] and also for reasons of cost-effectiveness, and for patient safety, biomarker-driven, personalized therapy choice is favoured over random or uniformized treatment decisions. The present results of the predictive value of CD69 marker seem, therefore, to be worth being validated in higher number of patients as a marker for the personalized choice of the appropriate class of biological therapy for the given RA patient.

A limitation of our study is the lack of the inclusion of an IL-6 nonresponder patient group, which would have made the study even more comprehensive.

In conclusion, Treg proportion is normalized in RA patients treated with long-standing anti-TNF or IL-6R blocker therapies. Our study examined a wide spectrum of T-cell subtypes and indicated that the remaining components of the T-cell pool are consistent with a permanent antigen-driven immunological process. In contrast to our former study with a short-term follow-up, we could observe a more complex alteration in the T-cell phenotype and that these changes are not much different among the particular anti-TNF agents—although the relatively low number of patients treated with each specific anti-TNF drug precludes firm conclusions in this regard. However, IL-6R blockade skews the adaptive immune system into a rather different profile, while the restoration of Treg proportion is an important common end-point during both therapies. CD4+CD69+ cell percentage is a potential candidate for the prediction of treatment response to anti-TNF therapy.

## Figures and Tables

**Table 1:** Clinical data and patient characteristics.

	Anti-TNF responders (*n* = 30)	Anti-TNF nonresponders (*n* = 19)	Anti-IL-6R responders (*n* = 43)	Newly diagnosed untreated RA (*n* = 19)
Mean age (range)	57.2 (26–75)	55.7 (29–71)	57.5 (34–76)	48.3 (22–67)^#^

Gender (female/male)	19/11	12/7	28/15	11/8

Anti-TNF treatments	30 (100.0)	19 (100.0)		
*n* (%) adalimumab	10 (33.3)	4 (21.1)		
Certolizumab	6 (20.0)	8 (42.1)		
Etanercept	7 (23.3)	4 (21.1)		
Infliximab	4 (13.3)	2 (10.5)		
Golimumab	3 (10.0)	1 (5.3)		

Mean disease duration (range)	11.3 (2–33) yrs	10.8 (4–44) yrs	12.0 (2–34) yrs	2.7 (1–3) months
Adalimumab	11.9 (3–24)	11.8 (5–19)		
Certolizumab	10.7 (2–31)	10.2 (4–44)		
Etanercept	12.4 (2–33)	11.0 (5–26)		
Infliximab	13.5 (4–22)	12.0 (5–19)		
Golimumab	8.0 (3–16)	9.0		

Mean duration of current biological therapy months (range)	29.8 (6–52)	30.95 (6–50)	33.0 (6–48)	—
Adalimumab	35.1 (6–52)	30.8 (24–48)		
Certolizumab	28.2 (8–42)	29 (6–43)		
Etanercept	26.7 (7–41)	36.8 (18–50)		
Infliximab	27.6 (10–33)	22.5 (8–37)		
Golimumab	31.4 (11–42)	41		

Prior use of TNF inhibitors	3/30	6/19	29/43	—
No. of switching,				
*n*: 1	3/3	4/6	15/26	
2	—	2/6	9/26	
3	—	—	5/26	

Patients on corticosteroid therapy *n* (%)	8 (26.7)	8 (42.1)	15 (40.5)	—
Adalimumab	3/10	1/4		
Certolizumab	1/6	3/8		
Etanercept	2/7	2/4		
Infliximab	2/4	1/2		
Golimumab	0/3	1/1		

Patients on conventional DMARD therapy *n* (%)	26 (86.7)	15 (79.0)	23 (62.2)^∗^	—
Adalimumab	8/10	3/4		
Certolizumab	5/6	6/8		
Etanercept	6/7	3/4		
Infliximab	4/4	2/2		
Golimumab	3/3	1/1		

ACPA positivity *n* (%)	24 (85.4)	14 (82.4)	21 (41.4)^∗^	19 (100.0)
Adalimumab	8/10	3/4		
Certolizumab	5/6	6/8		
Etanercept	5/7	2/4		
Infliximab	3/4	2/2		
Golimumab	3/3	1/1		
DAS 28 score (mean ± SD)	2.2 ± 0.8	5.12 ± 1.29^+^	1.89 ± 0.87	7.71 ± 4.06^#^
Adalimumab	2.16 ± 0.79	4.64 ± 1.35		
Certolizumab	2.09 ± 1.18	5.53 ± 0.82		
Etanercept	1.70 ± 0.48	5.18 ± 1.98		
Infliximab	3.00 ± 0.92	4.95 ± 0.27		
Golimumab	2.36 ± 0.47	4.21 ± 0.00		

^∗^ *p* < 0.05 between anti-TNF responders and IL-6R blocker responders. ^+^*p* < 0.05 between anti-TNF nonresponders and IL-6R blocker responders, ^#^*p* < 0.05 between newly diagnosed untreated RA group and all other groups. DMARD: disease-modifying antirheumatic drug; ACPA: anti-citrullinated protein antibody; DAS28: disease activity score with 28 joints; TNF: tumor necrosis factor alpha; IL-6R: interleukin-6 receptor.

**Table 2:** Prevalence values of the various T-cell subsets in the examined patient groups and controls.

		CD4	CD4/CD45RA	CD4/CD45RO	CD4/CD69	CD4/HLADR	CD4/CD25	CD8	CD8/CD45RA	CD8/CD45RO	CD8/HLADR	CD8/CD69	Th1	Th2	Th17	Treg
Anti-TNF responders	Median	25.90	3.37	47.15	1.63	7.18	1.16	10.70	5.69	12.85	6.02	0.95	21.00	8.03	2.81	5.89
	25% percentile	17.43	1.54	20.68	0.46	4.44	0.56	5.39	2.27	5.173	2.63	0.46	16.70	7.14	2.25	4.34
	75% percentile	33.93	8.46	63.85	2.845	9.62	1.52	19.30	22.00	17.00	9.74	2.15	23.78	9.35	3.26	6.85

Anti-TNF nonresponders	Median	40.8^a^	4.13	53.90	1.31	4.33^a^	1.71	12.80	7.18	9.480	4.10	1.27	18.40	8.19	3.29	5.80
	25% percentile	24.50	2.55	22.10	1.07	2.39	1.09	6.00	2.08	3.550	1.20	0.59	6.90	6.740	1.41	4.64
	75% percentile	44.90	14.00	62.50	2.70	6.91	6.39	24.70	35.20	15.40	5.41	2.01	21.60	11.20	4.40	7.21

aIL-6R responders	Median	35.4^a^	3.02	40.00	4.14^a,b^	5.35	4.38^a^	5.71^a,b^	4.96	10.70	2.88	4.47^a,b^	5.48^a,b^	12.50^a^	5.01^a,b^	4.75
	25% percentile	26.6	1.52	8.620	1.46	3.32	1.50	4.00	2.01	7.370	1.64	2.25	3.04	8.540	3.95	4.08
	75% percentile	47.30	8.37	52.80	8.10	7.16	5.35	8.12	37.70	14.80	5.19	11.50	16.00	14.40	5.87	5.65

Newly diagnosed untreated RA patients	Median	43.9^a^	46.55^a,b,c^	48.60	2.72	3.47^a^	4.57^a^	12.40^c^	64.80^a,b,c^	28.25^a,b,c^	2.77	2.59^a,b^	12.15^a^	9.25	1.76^c^	3.14^a,b,c^
	25% percentile	37.05	34.90	37.25	2.36	2.97	3.19	9.78	57.48	21.05	1.84	1.77	10.20	8.84	1.61	2.81
	75% percentile	62.03	54.23	61.63	3.51	4.58	4.86	20.05	74.33	34.15	3.98	3.38	14.70	11.00	1.98	3.66

Healthy controls	Median	41.55^a^	52.65^a,b,c^	41.55	2.4	2.80^a,c^	7.13^a,b,c,d^	19.64^a,b,c^	69.20^a,b,c^	23.65^a,b,c^	2.3^a^	1.88^c^	10.75^a^	5.79^a,b,c,d^	1.04^a,b,c^	5.17^d^
	25% percentile	37.5	49.15	35.875	1.84	2.47	6.14	15.72	59.77	15.8	1.65	1.37	9.22	4.85	0.78	4.44
	75% percentile	43.65	62.638	49.4	2.85	3.88	7.79	24.12	75.77	28.2	2.92	3.08	12.37	7.09	1.19	5.91

Numbers indicate the median percentages and 25% and 75% percentile values within the whole T-cell population (CD4 and CD8 cells), or within CD4 cells (Th1, Th2, Th17, Treg, and ratios), or for the other markers within the CD4 or CD8 cells, as indicated. ^a^*p* < 0.05 versus anti-TNF responder, ^b^*p* < 0.05 versus anti-TNF nonresponder, ^c^*p* < 0.05 versus anti-IL-6 receptor responder, ^d^*p* < 0.05 versus newly diagnosed untreated RA patients. TNF: tumor necrosis factor alpha; IL-6R: interleukin-6 receptor.

**Table 3:** Subgroup analysis in the long-term-treated RA patient groups according to the length of the biological therapy.

			CD4	CD4/CD45RA	CD4/CD45RO	CD4/CD69	CD4/HLADR	CD4/CD25	CD8	CD8/CD45RA	CD8/CD45RO	CD8/HLADR	CD8/CD69	Th1	Th2	Th17	Treg
Anti-TNF responders	“Short”	Median	25.35	4.60^a^	46.95	1.95	8.03	1.00	9.18	4.68^a^	5.97	2.90	0.56	18.2	7.51	2.49	4.67
		25% percentile	32.47	7.73	60.32	2.74	9.13	1.62	19.45	46.70	18.10	10.51	2.50	23.15	9.02	3.35	6.82
		75% percentile	25.35	4.60	46.95	1.95	8.03	1.00	9.18	4.68	5.97	2.90	0.56	18.2	7.51	2.49	4.67
	“Mid”	Median	31.25	8.14^a^	49.25^a^	1.92	5.58	1.16	13.05	14.11^a^	15.25	4.01	1.10	20.7	7.76	2.90	5.57
		25% percentile	26.65	4.06	45.45	1.32	5.36	0.88	10.29	5.67	12.22	1.84	0.63	18.8	7.17	2.56	4.88
		75% percentile	36.77	10.57	66.82	2.69	7.89	1.24	16.07	31.45	17.95	5.96	1.62	23.47	10.05	3.35	6.11
	“Long”	Median	22.20	1.79	37.30	0.84	6.02	1.10	8.57	3.97	10.85	6.82	0.78	19.90	8.06	2.38	6.43
		25% percentile	16.70	1.28	13.69	0.23	4.35	0.51	4.63	1.08	4.36	3.03	0.49	16.65	6.99	2.17	4.30
		75% percentile	32.87	2.91	56.00	2.59	12.57	1.77	17.97	6.32	14.20	9.74	1.09	23.77	9.00	3.04	6.86

Anti-TNF nonresponders	“Short”	Median	41.00	3.49	53.20	1.07	5.89	1.59	9.54	2.08	5.72	5.41	0.61	18.70	7.96	4.93	4.44
		25% percentile	30.90	3.24	26.80	1.03	3.78	0.97	7.62	1.55	3.16	2.82	0.58	12.79	7.51	2.85	4.32
		75% percentile	41.30	10.99	57.85	3.02	7.25	5.52	18.87	11.69	16.11	9.35	0.62	20.45	11.23	5.12	5.70
	“Long”	Median	40.15	4.52	55.70	1.37	4.20	2.32	13.35	7.45	11.19	4.07	1.36	18.25	8.70	2.71	5.86
		25% percentile	25.62	2.47	22.92	1.15	2.60	1.16	6.33	2.40	6.86	1.33	1.05	11.92	6.65	1.45	5.45
		75% percentile	44.92	13.17	62.67	2.63	6.29	5.65	20.57	36.12	14.87	4.67	2.07	21.52	11.12	4.20	7.81

Anti-IL-6R responders	“Short”	Median	31.70	3.96	28.7	5.19	4.17	4.28	5.79	3.47	8.52	2.88	3.68	4.83	13.00	5.19	4.69
		25% percentile	25.30	1.81	17.7	1.85	2.52	1.50	3.73	1.71	7.37	1.46	1.76	3.40	9.31	3.09	4.01
		75% percentile	42.40	8.03	42.7	8.10	5.83	4.78	6.96	9.96	15.20	4.23	11.50	13.60	14.30	6.19	5.83
	“Mid”	Median	27.05	3.21	47.75	3.63	5.65	4.30	5.41	4.54	9.70	2.92	6.96	7.41	11.21	4.69	4.75
		25% percentile	25.77	2.51	6.41	1.35	3.51	1.07	4.04	2.89	8.15	1.60	3.37	3.44	6.74	3.27	4.58
		75% percentile	36.25	10.04	64.60	6.4	10.47	6.02	8.82	32.48	12.30	8.22	10.29	17.82	16.40	5.38	5.00
	“Long”	Median	41.35	2.89	43.35	4.10	5.38	4.71	5.53	5.83	11.70	2.81	3.99	5.20	11.90	4.96	4.63
		25% percentile	34.82	1.21	10.96	2.01	4.48	1.63	4.14	2.52	8.17	2.01	2.82	3.03	9.99	4.41	4.06
		75% percentile	49.07	4.86	51.02	9.30	7.39	5.34	7.68	39.12	14.27	4.51	12.35	8.77	13.40	5.43	6.32

Numbers indicate the median percentages and 25% and 75% percentile values within the whole T-cell population (CD4 and CD8 cells), or within CD4 cells (Th1, Th2, Th17, Treg, and ratios), or for the other markers within the CD4 or CD8 cells, as indicated. ^a^*p* < 0.05 versus anti-TNF responders long-term.

**Table 4:** Subgroup analysis of cell prevalences in the long-term-treated RA patient groups according to the number of previous switches of anti-TNF therapies.

			CD4	CD4/CD45RA	CD4/CD45RO	CD4/CD69	CD4/HLADR	CD4/CD25	CD8	CD8/CD45RA	CD8/CD45RO	CD8/HLADR	CD8/CD69	Th1	Th2	Th17	Treg
Anti-TNF responders	“Switched”	Median	33.40	14.10	40.30	1.47	5.36	0.96	14.20	20.20	14.10	5.51	0.96	16.70	8.95	2.67	6.84
		25% percentile	33.05	8.08	40.10	1.14	4.57	0.69	12.40	12.18	9.26	4.07	0.57	15.95	8.36	2.30	5.51
		75% percentile	41.75	18.55	44.80	2.02	5.44	1.21	16.50	25.60	15.25	5.75	1.49	17.55	9.47	2.92	6.87
	“Nonswitched”	Median	24.40	3.17	47.30	1.80	7.92	1.20	9.77	5.41	12.60	6.19	0.86	21.70	8.02	2.82	5.85
		25% percentile	17.25	1.50	19.65	0.46	4.49	0.58	5.34	2.19	5.60	2.89	0.48	17.20	7.13	2.25	4.44
		75% percentile	33.15	7.77	64.80	2.91	9.84	1.57	19.25	15.50	17.20	9.74	2.09	23.85	9.24	3.30	6.63

Anti-TNF nonresponders	“Switched”	Median	30.15	2.77	38.55	1.42	3.59	1.54	24.75	8.55	11.02	2.86	1.85	20.90	8.81	1.40	7.09
		25% percentile	19.07	2.11	6.30	1.28	1.85	0.94	11.11	2.22	2.76	0.96	1.31	15.40	8.017	1.11	6.19
		75% percentile	41.37	11.25	58.70	2.21	6.38	3.53	27.35	29.35	13.57	4.95	2.53	21.90	10.31	3.48	9.19
	“Nonswitched”	Median	41.00	4.92	57.50	1.25	4.33	2.94	9.96	7.18	9.48	4.31	1.05	18.10	7.07	4.11	5.65
		25% percentile	29.80	3.49	49.80	1.07	2.69	1.19	6.00	2.17	5.72	1.38	0.53	6.90	6.39	2.08	4.64
		75% percentile	45.00	12.90	62.50	2.70	6.08	6.39	17.30	33.80	15.40	5.41	1.49	21.50	12.90	4.40	6.14

Anti-IL-6R responders	“Nonswitched”	Median	41.40	1.88	40.00	5.51	5.35	4.50	6.13	4.96	11.50	2.15	5.09	3.90	12.80	5.20	4.85
		25% percentile	31.30	1.34	21.20	3.40	3.43	2.35	4.72	2.39	8.49	1.47	2.96	3.00	9.93	4.38	4.12
		75% percentile	52.25	7.79	58.10	12.76	7.62	5.50	7.54	37.95	15.20	3.41	12.70	7.67	14.55	5.815	5.98
	Switching once	Median	25.70	5.43	28.70	1.44	5.55	1.50	7.54	3.37	8.20	4.23	2.45	16.00	10.70	3.95	4.69
		25% percentile	22.90	1.60	9.04	0.77	3.97	0.66	3.86	1.87	5.97	2.30	0.92	6.12	7.35	3.07	4.39
		75% percentile	33.65	10.05	43.45	1.85	7.16	3.94	10.2045	33.90	11.75	5.68	5.24	17.85	12.20	4.89	6.17
	Switching twice	Median	35.40	4.12	12.50	4.60	5.83	5.14	6.36	6.64	12.50	4.28	4.11	5.65	13.30	5.41	4.45
		25% percentile	26.60	3.02	10.70	3.13	5.77	2.47	4.17	2.47	10.70	2.88	3.12	4.92	7.85	3.73	4.21
		75% percentile	48.90	10.60	13.30	10.60	8.88	6.57	9.65	37.70	13.30	9.37	9.97	17.30	15.80	6.45	4.75
	Switching three times	Median	34.30	2.58	11.80	5.00^a^	4.17	4.92	5.35	5.93	10.50	2.29	10.40^a^	3.00^a^	12.70	5.07	4.81
		25% percentile	27.50	2.38	3.29	4.14	3.11	4.78	5.02	5.63	6.46	1.98	5.04	2.72	12.50	5.01	3.82
		75% percentile	42.40	2.89	50.00	15.80	4.68	5.35	5.74	9.96	14.80	3.80	16.30	4.33	13.00	5.30	5.06

Numbers indicate the median percentages and 25% and 75% percentile values within the whole T-cell population (CD4 and CD8 cells) or within CD4 cells (Th1, Th2, Th17, Treg, and ratios), or for the other markers within the CD4 or CD8 cells, as indicated. ^a^*p* < 0.05 versus anti-IL-6R responders, switching once.

**Table 5:** Prevalence values of the various T-cell subsets according to different anti-TNF therapies.

		CD4	CD4/CD45RA	CD4/CD45RO	CD4/CD69	CD4/HLADR	CD8	CD8/CD45RA	CD8/CD45RO	CD8/HLADR	CD8/CD69	CD4/CD25	Th1	Th2	Th17	Treg
Infliximab	Median	37.20	5.11	43.80	2.57	4.57	14.35	6.09	5.25	4.94	0.72	0.76	8.97	7.17	2.47	5.810
	25% percentile	26.55	1.83	22.00	0.85	3.18	8.89	1.98	4.21	3.66	1.00	0.97	18.40	8.22	1.83	4.590
	75% percentile	47.20	8.74	50.20	5.22	6.76	19.53	19.83	12.18	6.96	1.90	3.21	19.55	10.53	4.24	7.128

Adalimumab	Median	29.45	3.24	51.15	1.50	6.02	14.10	4.78	11.29	5.20	0.46	0.50	16.65	7.34	3.01	5.665
	25% percentile	16.71	2.07	30.04	0.85	3.86	5.675	1.60	5.010	1.67	0.88	1.01	21.00	8.24	2.26	4.273
	75% percentile	37.03	10.42	62.28	2.37	10.02	23.55	6.44	15.75	9.78	1.75	2.07	23.40	9.72	4.17	7.318

Etanercept	Median	32.70	4.29	67.20	1.88	7.86	9.36	11.80	13.80	6.19	0.68	1.19	5.25	6.34	3.29	4.960
	25% percentile	22.62	1.79	53.70	0.57	4.45	4.44	3.77	9.30	1.82	1.16	1.69	21.00	8.19	1.82	3.890
	75% percentile	40.14	7.75	77.10	3.04	13.3	24.80	36.30	21.90	10.60	1.42	5.49	23.00	11.30	3.98	5.930

Certolizumab	Median	27.30	4.91	39.30	1.20	4.91	12.35	9.11	10.88	3.36	0.48	1.18	16.93	6.79	2.31	6.600
	25% percentile	16.34	2.04	0.79	0.57	1.61	7.188	1.15	0.89	1.05	2.04	1.50	19.75	8.03	1.40	5.645
	75% percentile	44.93	17.05	62.20	2.78	8.81	19.98	39.80	19.80	6.56	2.68	3.39	22.20	9.60	3.45	8.260

Golimumab	Median	24.09	2.64	35.50	0.83	5.72	6.76	6.29	14.30	7.14	0.23	0.58	16.80	6.00	2.66	6.765
	25% percentile	18.75	0.62	5.710	0.16	4.65	2.12	1.72	3.79	3.91	0.68	1.12	23.55	6.68	1.67	6.008
	75% percentile	44.08	11.61	55.45	2.29	9.32	12.42	16.95	16.15	14.79	1.71	1.44	30.53	9.28	3.15	6.923

Numbers indicate the median percentages and 25% and 75% percentile values within the whole T-cell population (CD4 and CD8 cells), or within CD4 cells (Th1, Th2, Th17, Treg, and ratios), or for the other markers within the CD4 or CD8 cells, as indicated.
